# Preliminary data on the vector competence of *Aedes caspius* for* Dirofililaria immitis* in a traditionally endemic area of northern Italy

**DOI:** 10.1186/s13071-025-06828-6

**Published:** 2025-06-05

**Authors:** Alice Vismarra, Marco Genchi, Alessia Maltoni, Manuela Semeraro, Laura Helen Kramer, Mattia Calzolari, Annalisa Grisendi, Gastone Dalmonte, Marta Fozzer

**Affiliations:** 1 Department of Veterinary Medicine Sciences, University of Parma, Strada del Taglio, 10, 43126 Parma, Italy; 2 Istituto Zooprofilattico Sperimentale della Lombardia ed Emilia-Romagna, Reggio Emilia, Italy

**Keywords:** *Dirofilaria immitis*, *Aedes caspius*, *Culex pipiens*, *Aedes albopictus*

## Abstract

**Background:**

*Dirofilaria immitis*, the agent of canine and feline heartworm disease, and *Dirofilaria repens*, the agent of subcutaneous dirofilariosis, are widespread mosquito-borne helminths. The present study is aimed at updating current knowledge of the composition of potential vector species in the northern region of Emilia-Romagna, a traditionally endemic area for *D. immitis* and *D. repens*.

**Methods:**

Mosquitoes were collected in 2022 and 2023 as part of the regional surveillance plan for West Nile Virus (WNV). The capture zones included peri-urban and rural areas and mosquitoes were captured with CDC-CO_2_ traps. DNA from approximately 30% of female mosquitoes of each captured species (*Culex pipiens*, *Aedes caspius*, *Aedes vexans*, *Aedes albopictus*) was extracted and analyzed for the presence of *D. immitis* and *D. repens.*

**Results:**

A total of 140 pools (~ 20 mosquitoes each) in 2022 and 133 in 2023 have been analyzed. DNA of *D. immitis* was identified in 14 pools in 2022 and in 15 pools in 2023. None of the pools was positive for *D. repens*. In 2022, about 85% of the positive pools belonged to *Ae. caspius* species (11/13) and the other three pools to *Ae. vexans*. In 2023, 73% of the positive pools belonged to *Ae. caspius*, followed by *Ae. vexans* and *Ae. albopictus* (both 13.3%). A significant overlap emerged from the same traps positioned in Ferrara and Bologna provinces, which tested positive for *D. immitis* in both 2022 and 2023.

**Conclusions:**

These data highlight how, despite the abundance of *Cx. pipiens* captured, the most receptive species for *D. immitis* appear to be *Ae. caspius* and *Ae. vexans*. Furthermore, the geographical data highlights how the areas of the province of Ferrara and Bologna are the main geographical reservoirs of the parasite.

**Graphical abstract:**

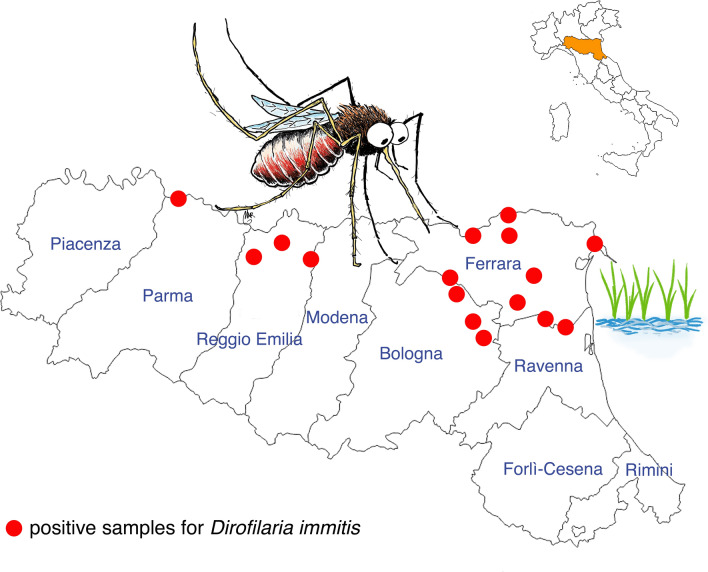

## Background

*Dirofilaria immitis*, the agent of canine and feline heartworm disease (HWD), is a widespread mosquito-borne helminth. The distribution of *D. immitis* is expanding from traditionally endemic areas of southern Europe to areas of central and northern Europe that were, until recently, considered free of infection [1].

For example, it has been reported that infection prevalence of *D. immitis* in dogs has decreased in recent years in northern Italy but is spreading to previously unaffected areas of central and southern Italy [2, 3]. While practitioners’ awareness of the disease and the use of preventive measures (chemoprophylaxis with macrocyclic lactones) are widespread in northern Italy, they are less so in central and southern areas, allowing the spread of infection. However, two recent surveys reported that veterinary practitioners in northern Italy continue to diagnose the infection in dogs, with practices from several provinces reporting over 20 cases of infection in the 12 months preceding the surveys [4, 5].

Essential prerequisites for heartworm transmission include the presence of competent mosquito vectors and a climate that provides adequate temperature and humidity to support the mosquito population and can also sustain sufficient heat to allow maturation of ingested microfilariae into infective, third-stage larvae (L3) within the vector. The ubiquitous presence of one or more species of vector-competent mosquitoes makes transmission possible wherever a reservoir of infection and favorable climatic conditions coexist [6].

There are over 60 mosquito species that potentially transmit *D. immitis*, with *Aedes*, *Anopheles*, and *Culex* among the most common genera [7]. Currently available data, though scarce and dated, suggests that *Culex pipiens*, *Aedes caspius*, and *Aedes albopictus* are among the main vector species for *D. immitis* in northern Italy [8]. The present study is aimed at updating current knowledge of the composition of competent vector species in the northern region of Emilia-Romagna along the Po River valley, a traditionally endemic area for *D. immitis*.

## Methods

### Sample collection

**Fig. 1:**
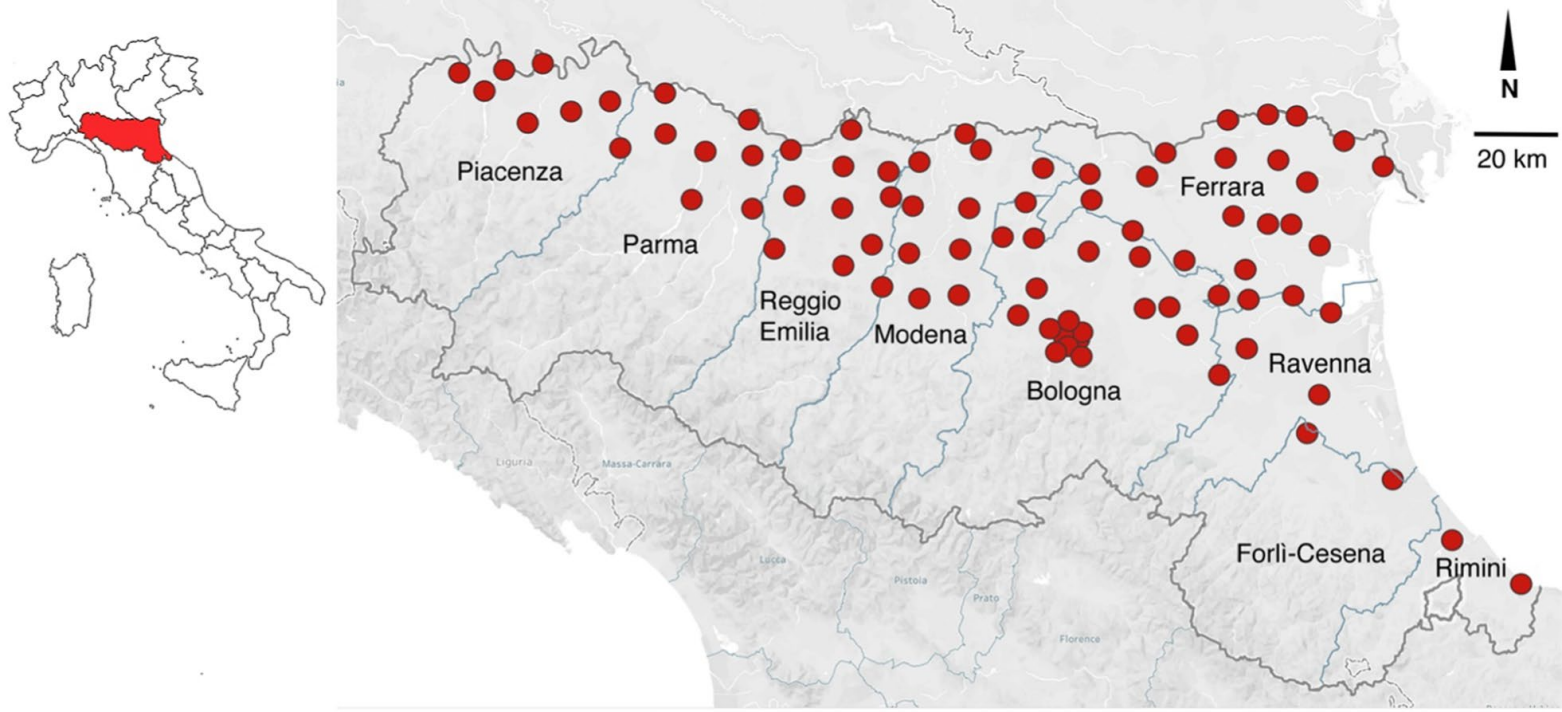
Mosquito capture areas in the different provinces of the Emilia-Romagna region (figure created with Tableau Public online software available at: https://public.tableau.com/app/discover)

Mosquitoes were collected in 2022 and 2023 in the Emilia-Romagna region (northern Italy) as part of the regional surveillance plan for West Nile Virus (WNV) [9]. The capture areas included mainly peri-urban and rural areas. Figure 1 presents a map of Italy, divided into regions, to show the position of the Emilia-Romagna region where the study was carried out. This region is then reported in larger format to indicate where mosquito traps were positioned in the different provinces. Mosquitoes were captured using US Centers for Disease Control and Prevention (CDC) CO_2_ traps, according to a previous study [10]. The mosquitoes analyzed in the present study were those captured from August 17 to August 26, 2022, and August 17 to August 24, 2023. The choice of this sampling period was based on the likelihood of finding infected mosquitoes and on data obtained previously in our laboratory.

Captured mosquitoes were placed at −20 °C and then sorted by capture area, sampling date and species, that were identified following the dichotomous keys published by Becker et al. [11]. Differentiation between *Cx. pipiens* and *Culex torrentium* was not carried out, based on published data indicating that *Cx. torrentium* is not present in the Emilia-Romagna region [12].

Approximately 30% of female mosquitoes from each species from each capture area were pooled into groups of about 20 mosquitoes/pool. Molecular analyses for DNA of *D. immitis* and *D. repens* identification were then carried out. When a pool was found to be positive for *Dirofilaria* spp. DNA, further pools from the same traps were analyzed to include all captured mosquitoes from the area.

### DNA extraction

DNA extraction was performed using the automated Maxwell^®^ system (Promega, WI, USA), utilizing specific DNA RSC Tissue DNA cartridges. A pre-digestion was applied by mechanical and chemical lysis. After adding 200 µl of PBS in each Eppendorf tube containing 20 female mosquitoes each (pool), mechanical lysis was carried out with a pellet pestle for about 10 min. Then, 300 µl of tissue lysis buffer (TLA) and 30 µl of proteinase K (Promega, WI, USA) were added, and to complete the digestion, pools were vortexed for 10 s and then placed in an Eppendorf ThermoMixer^®^ (Eppendorf, Hamburg, Germany) at 56 °C for about 1.5 h. Subsequently, about 400 µl of the homogenate obtained from the previous digestion was introduced into specific cartridges for DNA extraction. DNA was eluted in 100 µl of elution buffer and quantified using a spectrophotometer (BioSpectrometer^®^, Eppendorf, Hamburg, Germany). Samples with high genetic material concentration were diluted 1:10, then submitted to amplification through real-time polymerase chain reaction (PCR).

### Real-time PCR

Real-time PCR was conducted to detect the presence of *Dirofilaria repens* and *D. immitis*, following the protocol of Sulesco et al. [13], with some modifications (for example, the annealing temperature was set at 51 °C instead of 61 °C as cited in the original paper, and the number of cycles was reduced to 45 instead of 55). A fragment of the cytochrome c oxidase subunit 1 (*COI*) gene of *D. repens* was amplified using the primers RepF (5′-GAGATGGCGTTTCCTCGT-3′) and RepR (5′-GACCATCAACACTTAAAG-3′) along with the probe RepT (5′ JOE-GTTGCTTTGTTAATGGTTTATC-BHQ1-3′) [JOE = 6-carboxy-4′,5′-dichloro-2′,7′-dimethylfluorescein, BHQ1 = black hole quencher 1]. For *D. immitis*, a fragment of the 16S ribosomal RNA (rRNA) gene was amplified using the primers ImmF (5′-CTATATGTTACCTTAATTGG-3′) and ImmR (5′-CTTAACCATTATCTTAGATCAG-3′) along with the probe ImmT (5′ ROX-GTAGCTAGTAAGTTTACC TTG-BHQ2-3′) [ROX = 6-carboxy-x-rhodamine, BHQ2 = black hole quencher 2].

PCR was performed using the CFX96^®^ Real-Time System (Bio-Rad, CA, USA), first testing samples for *D. immitis* and then for *D. repens*. The reaction mixture had a final volume of 20 µl, consisting of 18 µl of mix (specific for *D. repens* or *D. immitis*) and 2 µl of DNA (diluted 1:10). The mix for *D. immitis* consisted of 10 µl of 2× SsoAdvanced Universal Probes Supermix (Bio-Rad, CA, USA), 0.8 pmol of ImmT probe, 4 pmol of ImmR, 4 pmol of ImmF, and 8.3 µl of H_2_O to reach the final volume. The mix for *D. repens* consisted of 10 µl of 2× SsoAdvanced Universal Probes Supermix (Bio-Rad, CA, USA), 16 pmol of RepT probe, 24 pmol of RepF, 24 pmol of RepR, and 8.5 µl of H_2_O to reach the final volume. Positive (DNA extracted from adult worms of *D. immitis* and *D. repens*) and negative controls (water) were added in each analysis. The thermal profile included an initial denaturation of 15 min at 95 °C followed by 45 cycles consisting of 15 s at 95 °C, 30 s at 51 °C, and an elongation of 30 s at 72 °C. The PCR ended with a final phase of 30 s at 40 °C. Samples were considered positive when the quantification cycle (C_q_) values ranged from 10 to 35 and doubtful with C_q_ ranging from 36 to 39.

### Statistical analyses

The minimum infection rate (MIR) [14] and the estimated infection rate (EIR) [15] were calculated using the following formulas:

$${\text{MIR}} = (x/n)*100\quad {\text{and}}\quad {\text{ERI}} = [1 - (1 - x/m)]^{1} /k*100,$$

where *x* is the number of positive pools, *n* is the total number of mosquitoes tested, *m* is the number of mosquito pools, and *k* is the average number of mosquitoes in each pool.

Comparison between the number of mosquitoes captured in 2022 and 2023 was made with analysis of covariance (ANCOVA), while differences in *D. immitis*/*D. repens* infection were evaluated among mosquito species and province using the Student *t*-test and analysis of variance (ANOVA) test (GraphPad Prism^®^ version 10).

## Results

### Sample collection and identification of mosquitoes

A total of 8248 and 15,624 female mosquitoes were captured during the two capture periods considered in 2022 and in 2023, respectively (see Table 1). Captured species included *Cx. pipiens*, *Ae. caspius*, *Aedes vexans*, *Ae. albopictus*, *Coquillettidia richiardii*, and *Anopheles maculipennis* sensu lato (s.l.), and no statistically significant differences were highlighted comparing the number of captured mosquitoes vs. species vs. years (*P* = 0.36).

**Table 1:** Female mosquitoes captured during the two capture periods considered in 2022 and in 2023

Mosquito species	No. of female mosquitoes 2022	No. of female mosquitoes 2023
*Culex pipiens*	3569	6726
*Aedes caspius*	3178	5815
*Aedes vexans*	723	1320
*Aedes albopictus*	526	1628
*Coquillettidia richiardii*	203	29
*Anopheles maculipennis* s.l.	49	106
Total	8248	15,624

A total of 2800 mosquitoes (140 pools) from 2022 and a total of 2660 (133 pools) from 2023 were analyzed (Table 2). A further 36 pools were analyzed from several capture areas giving positive results at the initial screening (20 in 2022 and 16 in 2023). The geographical distribution highlighted that Ferrara province is certainly the area where the majority of *Ae. caspius* have been captured (*P* = 0.0007, *F* = 11.54, *R*^2^ = 0.9112). For the other most abundant species *Cx. pipiens* and *Ae. albopictus*, the distribution is similar among Reggio Emilia, Parma, Bologna, Ferrara, and Modena.

**Table 2:** Pools from 2022 and 2023 analyzed for *Dirofilaria* spp.

Species 2022	No. of pool (+ new selected)	Provinces							
		FC	MO	BO	RI	FE	RE	PR	RA
*Cx. pipiens*	41	2	10	13	1	11	2	0	2
*Ae. albopictus*	19 (+6)	0	6	0	3	6	7	3	0
*Ae. caspius*	64 (+9)	1	13	8	0	40	8	3	0
*Ae. vexans*	12 (+5)	0	0	3	0	6	8	0	0
*Cq. richiardii*	4	0	0	0	0	4	0	0	0
Total pools	140 (+20)	3	29	24	4	67	25	6	2

Species 2023	No. of pool (+ new selected)	Provinces						
		MO	BO	FE	RE	PR	RA	PC
*Cx. pipiens*	35	2	11	9	2	5	3	3
*Ae. albopictus*	24 (+5)	2	5	9	6	2	3	2
*Ae. caspius*	57	10	13	25	3	4	0	2
*Ae. vexans*	17 (+8)	4	10	5	1	2	0	1
*Cq. richiardii*	0 (+3)	0	3	0	0	0	0	0
Total pools	133 (+16)	18	33	42	13	13	6	8

FC: Forlì-Cesena; MO: Modena; BO: Bologna; RI: Rimini; FE: Ferrara; RE: Reggio Emilia; PR: Parma; RA: Ravenna

### *Dirofilaria immitis*- and *D. repens*-positive pools

**Fig. 2:**
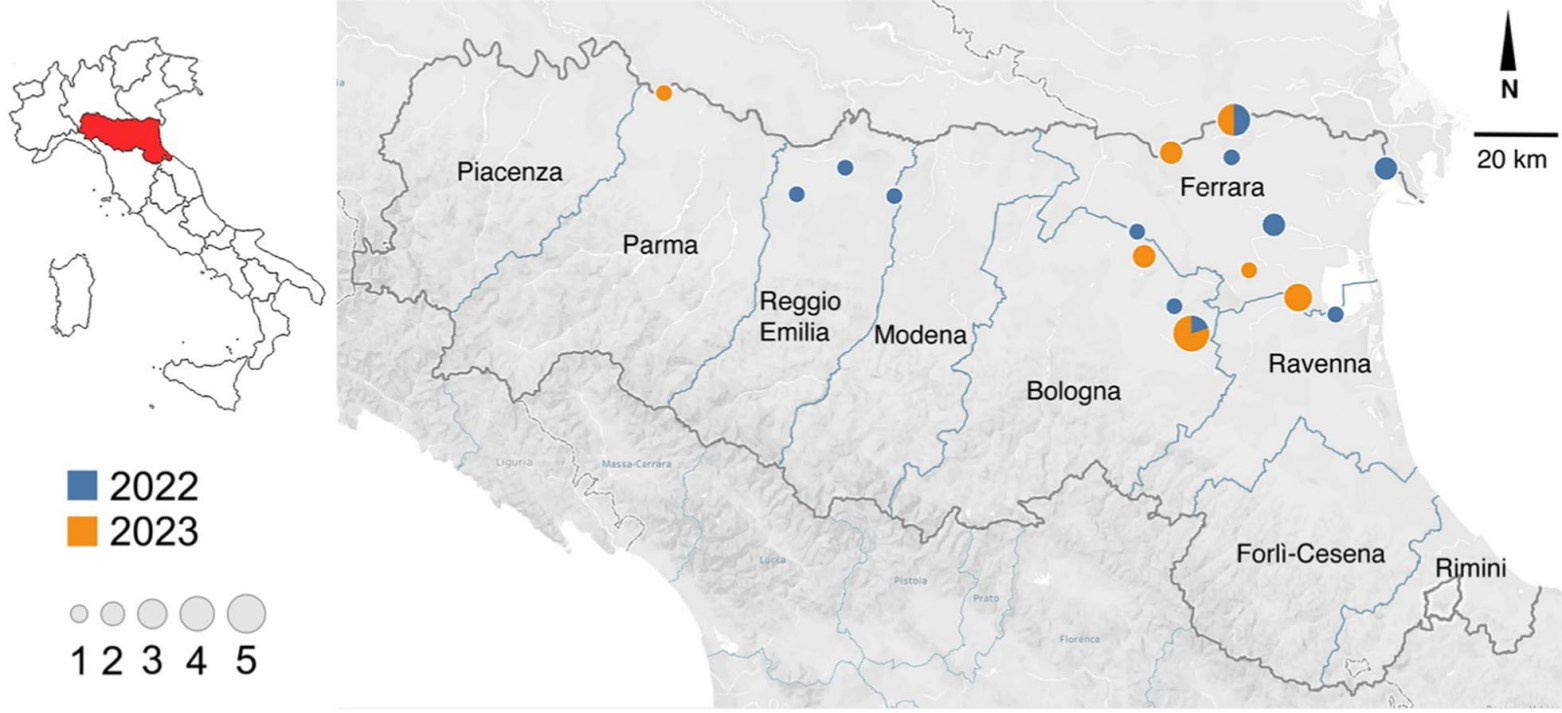
Mosquito pools positive for DNA of *D. immitis* captured in 2022 and in 2023 in the different provinces of the Emilia-Romagna region. Blue: positive pools in 2022; orange: positive pools in 2023 (figure created with Tableau Public online software available at: https://public.tableau.com/app/discover)

Figure 2 shows the location of *D. immitis*-positive pools for 2022 and 2023 in the different provinces of the Emilia-Romagna region. Results are detailed in Table 3. DNA of *D. immitis* was identified in 14 pools from 2022 and in 15 pools from 2023. Eleven pools of *Ae. caspius* were positive in 2022, the majority (9/11) of which were captured in the province of Ferrara, while two positive pools were from the provinces of Bologna and Reggio Emilia. Two pools of *Ae. vexans* captured in two different areas of the province of Reggio Emilia and one from Bologna were also positive. No captured mosquitoes were positive for *D. repens.*

**Table 3:** Mosquito pools positive for DNA of *D. immitis* captured in Emilia-Romagna in 2022 and in 2023

Positive pools	Species	Capture location	Province	Geographical coordinates	Year
2	*Ae. caspius*	Goro	Ferrara	44°51′47.2″N 12°17′22.7″E	Aug-2022
2	*Ae. caspius*	Riva del Po	Ferrara	44°57′58.8″N 11°50′04.7″E	
2	*Ae. caspius*	Ostellato	Ferrara	44°44′34.4″N 11°57′14.3″E	
1	*Ae. caspius*	Poggio Renatico	Ferrara	44°43′42.2″N 11°32′41.9″E	
1	*Ae. caspius*	Copparo	Ferrara	44°53′11.2″N 11°49′39.6″E	
1	*Ae. caspius*	Argenta	Ferrara	44°33′06.1″N 12°08′19.2″E	
1	*Ae. caspius*	Medicina	Bologna	44°30′39.7″N 11°42′26.4″E	
1	*Ae. caspius*	Novellara	Reggio Emilia	44°51′52.5″N 10°40′13.5″E	
1	*Ae. vexans*	Gattatico	Reggio Emilia	44°48′28.8″N 10°31′29.0″E	
1	*Ae. vexans*	Correggio	Reggio Emilia	44°48′15.3″N 10°49′02.6″E	
1	*Ae. vexans*	Molinella	Bologna	44°34′09.8″N 11°39′22.3″E	
1	*Ae. caspius*	Argenta	Ferrara	44°38′46.5″N 11°52′45.7″E	Aug-2023
3	*Ae. caspius*	Argenta	Ferrara	44°35′16.3″N 12°01′35.3″E	
1	*Ae. caspius*	Riva del Po	Ferrara	44°57′58.8″N 11°50′04.7″E	
2	*Ae. caspius*	Ferrara, near Po River	Ferrara	44°53′46.9″N 11°38′48.9″E	
4	*Ae. caspius*	Medicina	Bologna	44°30′39.7″N 11°42′26.4″E	
2	*Ae. vexans*	Baricella	Bologna	44°40′33.8″N 11°33′57.2″E	
1	*Ae. albopictus*	Riva del Po	Ferrara	44°57′58.8″N 11°50′04.7″E	
1	*Ae. albopictus*	Polesine Zibello	Parma	45°01′25.3″N 10°07′37.3″E	

Eleven pools of *Ae. caspius* were positive in 2023, four from the province of Bologna and seven from the province of Ferrara. Two pools of *Ae. vexans* (Bologna) and two pools of *Ae. albopictus* (Ferrara and Parma) were also positive. None of the analyzed pools was positive for *D. repens*.

Statistical analysis showed a significant difference among mosquito species positive for *D. immitis* when comparing *Ae. vexans* and *Ae. caspius* with other species that resulted negative for *D. immitis* DNA (*P* < 0.001, *F* = 80.73, *R*^2^ = 0.9838). Also the comparison between the two major species positive for the parasite DNA highlights a statistically significant difference (*Ae. caspius* vs. *Ae. vexans* *P* = 0.0034, *df* = 2, *t* = 17.000, 95% confidence interval (CI) = 6.35–10.65).

The following MIR values were obtained in 2022 vs. 2023: *Ae. caspius* 0.13% vs. 0.07%; *Ae. vexans* 0.04% vs. 0.013%; *Ae. albopictus* 0% vs. 0.013%. The EIR predictive value for *Ae. caspius* was 0.4% for both years, for *Ae. vexans* 0.1% vs. 0.075%, and for *Ae. albopictus* 0.075% in 2023.

## Discussion

The results from the present study show that *D. immitis* continues to circulate in mosquitoes in several areas of the Po River valley, a traditionally endemic area [16]. The eastern part of the valley, especially near the Adriatic coast, would appear to be a “hot spot,” and this is in agreement with the most recent surveys of veterinary practitioners who continue to diagnose the infection in dogs [4, 5]. No mosquitoes were positive for *D. repens*, again in agreement with current epidemiological data which suggest the parasite is rarely diagnosed in dogs from these areas [4, 5]. Interestingly, two traps positioned in the same areas (Riva del Po and Medicina [FE and BO, respectively, in Table 2]) in both 2022 and 2023 were positive for *D. immitis*, suggesting a stable circulation of the parasite. On the other hand, two traps that were positive in 2022 obtained negative results in 2023 (Reggio Emilia and Bologna), and a new area of ​​positivity was observed in 2023 in the province of Parma (see Fig. 2).

Results also suggest that *Ae. caspius* is an important potential vector of *D. immitis*, in agreement with several previous studies [8, 17–20]. This mosquito species, also called the rice field mosquito, is a floodwater species, passing the winters in colder climates as eggs that then emerge upon inundation in the spring. *Aedes caspius* exhibits aggressive behavior, readily feeds on humans and domestic animals, and can fly over 15 km from breeding sites [21]. A recent study carried out in the Po River valley reported that *Ae. caspius* was more abundant in the eastern and western parts of the valley [22], and a previous entomological survey carried out in the area of Ferrara, where 17/29 positive traps from the present study were placed, reported that *Ae. caspius* is the predominant species [10]. Rice fields are abundant, favoring the widespread diffusion of *Ae. caspius* in the region [23].

In the present study, *Ae. caspius* showed higher infection rates (MIR and EIR) than *Ae. vexans* and *Ae. albopictus*, and despite the low infection rates (MIR 0.07–0.13%/EIR 0.4%), it was the dominant species captured in most of the positive traps, therefore suggesting its possible role in transmission [8, 17, 24].

Interestingly, no *Cx. pipiens* were positive in the present study, despite it being an important vector for *D. immitis* in Europe [25]. However, in a previous study carried out in several regions of northeastern Italy, Latrofa et al. [8] reported that *Cx. pipiens* was approximately four times less infested than other species and significantly less infested than *Ae. caspius*, and the authors suggested that it is likely replacing *Cx. pipiens* as principal vector species for *D. immitis*. Furthermore, it must be kept in mind that *Cx. pipiens* predominates in urban areas, while our traps were placed in peri-urban and rural areas.

Both *D. immitis* and *D. repens* are zoonotic, and while cases of human subcutaneous dirofilariosis due to *D. repens* are increasing in Europe, human infection with *D. immitis* is uncommon [26]. Interestingly, the only two molecularly confirmed cases of human *D. immitis* infection in Italy were from patients residing in the areas under study here [27, 28]. As *Ae. caspius* has been reported as being strongly anthropophilic [10], our results suggest a potentially important zoonotic risk.

A limit to the present study is that nematode DNA can be found in both competent and non-competent mosquito vectors [29]. However, Rossi et al. [17] showed that among mosquitoes naturally infected with *D. immitis* through blood-feeding on microfilariemic dogs, vector efficiency was very high in *Ae. caspius*, which produced the highest number of infective larvae compared to other mosquito species.

The choice of only sampling those mosquitoes captured during late August, while not necessarily a limit to the study, is justified by the likelihood of finding infected mosquitoes and based on data obtained previously in our laboratory. Indeed, given the conditions needed for development, transmission, and circulation of *Dirofilaria* spp. (accumulation of heat developing units), mid-August was the optimal period to find captured mosquitoes harboring the parasite.

## Conclusions

In conclusion, *D. immitis* continues to circulate in mosquitoes in the Emilia-Romagna region of the Po River valley, despite the availability for over 30 years of effective chemoprophylaxis for the canine host [30] and the particular attention that veterinary practitioners have in recommending prevention [4, 5]. Interventions to control mosquito populations, including larvicidal and adulticidal treatment, remain challenging as they require proper planning, collaboration with farmers and communities, and the use of products that are safe for the environment. Since 2016 in the region of Emilia-Romagna, these interventions have been limited to tourist areas (and only when nuisance levels become very high) or when the circulation of vector-borne viruses has been reported [10]. Identification and control of the vector populations, along with continuous entomological surveys, can contribute to reducing the risk of infection in dogs as well as the human population.

## Data Availability

No datasets were generated or analyzed during the current study.

## Abbreviations

MIR: Minimum infection rate
EIR: Estimated infection rate
