# Correction: Preliminary data on the vector competence of *Aedes caspius* for *Dirofililaria immitis* in a traditionally endemic area of northern Italy

**DOI:** 10.1186/s13071-025-06895-9

**Published:** 2025-06-20

**Authors:** Alice Vismarra, Marco Genchi, Alessia Maltoni, Manuela Semeraro, Laura Helen Kramer, Mattia Calzolari, Annalisa Grisendi, Gastone Dalmonte, Marta Fozzer

**Affiliations:** 1 Department of Veterinary Medicine Sciences, University of Parma, Strada del Taglio, 10, 43126 Parma, Italy; 2 Istituto Zooprofilattico Sperimentale della Lombardia ed Emilia-Romagna, Reggio Emilia, Italy

**Correction: Parasites & Vectors (2025) 18:210** 10.1186/s13071-025-06828-6

Following publication of the original article [1], it was brought to the attention of the journal that, because of a processing error during production of the article, the article was published with the first names of the authors (erroneously) abbreviated. The names have since been fully spelled out in the published article.
